# Validation of the Manufacturing Methodology of Prestressed Fiber-Reinforced Polymer Concrete by the Variation of Process Parameters

**DOI:** 10.3390/ma16237377

**Published:** 2023-11-27

**Authors:** Michelle Engert, Kim Torben Werkle, Robert Wegner, Larissa Born, Götz T. Gresser, Hans-Christian Möhring

**Affiliations:** 1 Institute for Machine Tools (IfW), University of Stuttgart, 70174 Stuttgart, Germany; kim-torben.werkle@ifw.uni-stuttgart.de; 2 Institute for Textile and Fiber Technologies (ITFT), University of Stuttgart, 70569 Stuttgart, Germanygoetz.gresser@itft.uni-stuttgart.de (G.T.G.); 3 German Institutes of Textile and Fiber Research (DITF), 73770 Denkendorf, Germany

**Keywords:** composite, polymer concrete, manufacturing process

## Abstract

Polymer concrete has proved to be advantageous in machine building for many years thanks to its excellent damping properties. Until now, its use was limited to machine beds due to its comparatively low tensile strength. Its use in moving structural components has not been possible until now. Recent research results have shown that this challenge can be met by integrating prestressed carbon fibers. Until now, the production of samples out of prestressed fiber-reinforced polymer concrete has been carried out according to fixed specifications. It is not yet clear whether these specifications are suitable to fully exploit the potential of the material. Samples manufactured to these specifications show at least a large scatter in bending stiffness. Within the scope of this paper, the existing manufacturing process is validated by the variation of process steps. Specifically, this involved the use of a shaker, variation of the dwell time in the mold, variation of the resin content, and the procedure for impregnating the fibers. The characterization of the samples showed that the scatter could only be reduced by increasing the dwell time. However, this leads to a decrease in bending stiffness and, thus, is not suitable for further improvement of the novel material.

## 1. Introduction

To achieve the objectives of enhancing precision in machine tool manufacturing, the utilization of innovative materials has become increasingly necessary. The key attributes to be taken into consideration are a great vibration damping and temperature stability. Polymer concrete, also referred to as mineral cast, is a composite material composed of a thermo-setting matrix material like epoxy resin or polyester and mineral fillers. This material exhibits a low density, an exceptional damping behavior, favorable thermal properties [1], and has a low CO_2_ equivalent [2]. The combination of these properties makes polymer concrete an ideal material to be implemented in machine tool design. Since the 1970s, it has been employed in the form of machine beds [1]. Moreover, it is used to fill machine structures to enhance the dynamic behavior of the components [3,4]. There have been attempts to utilize the material for frame structures as well, but polymer concrete frames show nearly twice the amount of deformation under the same load compared with cast iron frames [5]. This discrepancy contradicts the objectives of machine tool design to enable the production of increasingly accurate components. The notable deformation of the composite material is primarily attributed to its relatively low tensile strength [6] and its high tendency to creep [7]. It is well known from the literature that the mechanical properties of polymer concrete are highly dependent on the material composition. Specifically, the matrix proportion as well as the quantity and type of fillers, including fly ash, are significant factors [8,9]. Furthermore, it has been shown that the mechanical properties of polymer concrete can be influenced by the addition of different fibers [8,10,11]. Short-fibered carbon should be mentioned here which has been added to improve the flexural and compressive properties, as indicated by several studies [12,13]. To further improve the mechanical properties, the carbon rovings can be prestressed, resulting in an approximate 150% enhancement in the stiffness of polymer concrete [14]. All previous tests conducted on the novel material have demonstrated a significant scatter in the results [14]. Therefore, the parameters of the previous manufacturing process, which has not been investigated in detail so far, were deliberately adjusted in this study to identify possible avenues for minimizing the scatter in the experimental results. Thus, for the first time, the manufacturing process of prestressed fiber-reinforced polymer concrete is considered in this paper.

## 2. Materials and Methods

### 2.1. Sample Preparation

The present study was aimed at investigating the effects of process parameters on the reproducibility of the bending properties of fiber-reinforced and prestressed fiber-reinforced polymer concrete. Within this section, the usual workflow for samples made out of this novel composite material is explained. 

The starting material for this hybrid material was the self-compacting polymer concrete EPUMENT 130/3, manufactured by RAMPF Machine Systems (Wangen, Germany). Currently, this material is utilized for filling cast structures to enhance vibration damping [6]. Test samples with dimensions of 50 × 50 × 500 mm^3^ were reinforced at five points, each of which contained bundles of six 24 k carbon rovings (GRAFIL 34-700, 1600 tex), manufactured by Mitsubishi Chemical Carbon Fiber and Composites (Sacramento, CA, USA). The fiber volume fraction was 1.1%. The test samples were designed based on a simplified depiction of a long machine arm of a machine tool.

Aluminum molds were used to manufacture the samples. A prestressing force of 3000 N was applied, which was equivalent to a tensile stress of around 108 MPa, using specially developed prestressing mechanisms [15]. The force was continuously monitored throughout the 24 h curing process using two load cells (type: KM26-10kN; manufacturer: ME-Meßsysteme GmbH, Hennigsdorf, Germany) integrated in the mold. No adjustments to the prestressing force were made during the process.

During the casting process, strain gauges (type: 6/120ZE LY41; manufacturer: HBM) were integrated into some of the test samples. These strain gauges were placed on one of the carbon roving bundles in the lower reinforcement plane at a distance of 100 mm to the edge of the mold. Additionally, PT100 temperature sensors were positioned inside the polymer concrete matrix adjacent to the strain gauges. The cables of the sensors were led out at approximately half the length of the sample.

**Figure 1:**
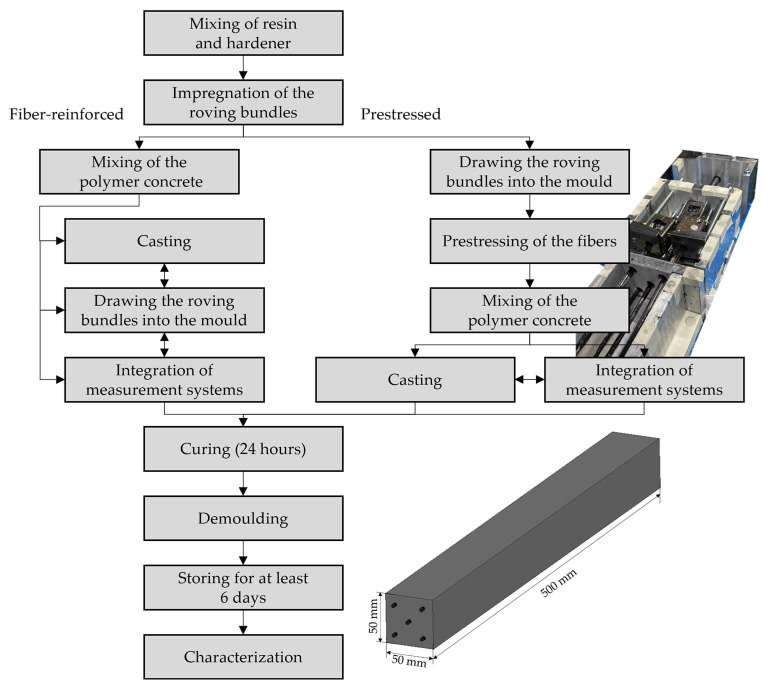
Workflow for the production of fiber-reinforced and prestressed fiber-reinforced polymer concrete.

The manufacturing process adhered to strict specifications (see Figure 1). In the initial step, the resin and hardener were mixed according to the manufacturer’s instructions. The fluid epoxy resin was then used for the impregnation of the carbon roving bundle. This involved dipping the roving bundles into the resin-hardener mixture and, subsequently, removing any excess resin. For the production of prestressed samples, the impregnated roving bundles were immediately drawn into the mold, followed by the application of the prestressing force. In the final step, the epoxy resin was mixed with mineral fillers, and the polymer concrete mixture was filled into the mold. During this step, measuring systems could be integrated into the samples. A different procedure was followed for the production of fiber-reinforced samples. After the carbon roving bundles were impregnated and the polymer concrete was mixed, a layer of polymer concrete was initially placed in the mold. Subsequently, the roving bundles of the lowest reinforcement layer were drawn into the mold with previously inserted roving bundles manually tensioned to ensure their straight alignment within the mold. After a minimum of 24 h since the mixture of resin and hardener, all samples were demolded and stored at room temperature (test conditions 22 °C at 38% humidity) for at least 6 days. 

### 2.2. Experimental Set-Up

**Figure 2:**
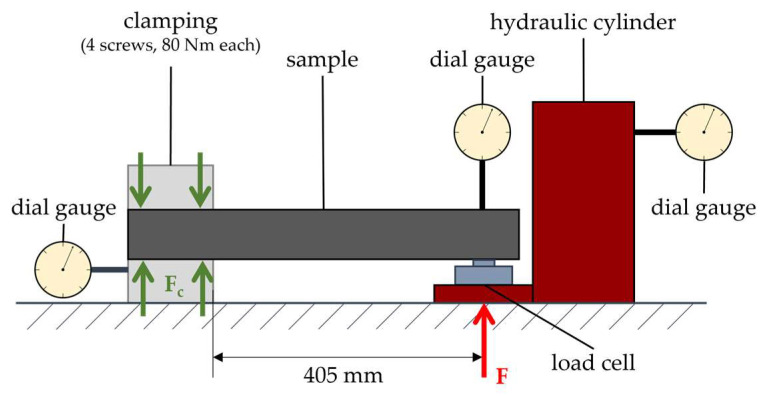
Experimental setup for the bending tests.

To characterize the material properties, uniaxial bending tests were carried out, replicating the static load conditions experienced by a machine arm of a milling machine. The experimental set-up is shown in Figure 2. The sample was fastened to the fixture with four screws. A load cell of type KM26-10kN, manufactured by ME-Meßsysteme GmbH, was employed to measure the force at a distance of 405 mm to the clamping device. The force was applied using a hydraulic cylinder. During the experiment, the deflection of the sample was measured with a dial gauge. Additionally, two more dial gauges were utilized to monitor the position of the hydraulic cylinder and the clamping device. Prior to the bending tests, the integrity of the test set-up was verified by performing a validation using two steel bars of different thicknesses.

### 2.3. Manufacturing Influences on the Mechanical Properties of Polymer Concrete

To identify methods for mitigating the large scatter of the bending stiffness observed in prior tests [14], this study used insights gained from analyses of pure polymer concrete. It was noted that the existence of cavities could compromise the mechanical characteristics of the base material, among other factors [16]. The uneven occurrence and distribution of these factors could account for the large scatter observed in the results. One approach to minimize the cavities is to use a shaker during the curing process to compact the mineral particles homogeneously [17]. To implement this, the fiber-reinforced samples were subjected to 20 min of shaking at 35 Hz immediately after filling the mold. Another strategy for reducing cavities is to increase the flowability of the mineral casting and, thus, the potential outgassing capability by raising the resin content [17]. However, it is well documented in the literature that this often leads to a decrease in the compression and bending properties [18]. To assess the impact of material viscosity, a test sample batch was produced with a 0.5% increase in resin content.

**Figure 3:**
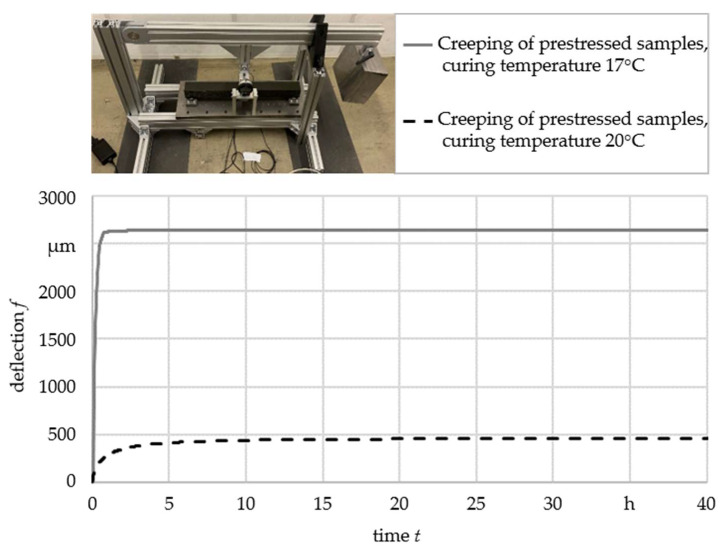
Creep of prestressed fiber-reinforced polymer concrete after curing at different temperatures.

Another influence on the material characteristics of pure polymer concrete is represented by the curing temperature and the dwell time in the mold [19]. In creep tests on a 3-point bending test rig with a permanent load of 675 N, it was shown that also prestressed fiber-reinforced polymer concrete had a curing temperature-dependent material behavior (see Figure 3). The tests were carried out on two test samples per temperature. For clarity reasons, only the deflection of one sample at each temperature was plotted. The diagram illustrates that even a minor rise in curing temperature by 3 K can decrease the deflection of the test samples caused by the creep behavior of polymer concrete by over 80%. The exact reasons for this material behavior are not yet clear, but it was assumed that the curing time decreases with increasing curing temperature. For this reason, the behavior of the test samples was examined after different curing times within the mold.

In addition to the previously mentioned tests, a subset of the samples was prepared with variations in the manufacturing processes. This allowed us to investigate the influences of the smallest variations in the manufacturing process of the samples. Additionally, the test samples varied with regard to the prestressing force of the integrated carbon fiber rovings. The purpose here was to investigate whether the application of a minor prestressing force affected the reinforcement structure layer in the fiber-reinforced test samples. In the case of prestressed test samples, the investigation was aimed at determining whether applying a lower prestressing force could enhance process reliability and, consequently, reduce scatter in bending stiffness test results. The resulting test plan is presented in Table 1. Each variant was tested using a minimum sample size of three. Initially, the bending properties were determined for each sample, followed by the creation of longitudinal sections using a diamond saw blade. To produce the cross-sections, the samples were initially sliced in half lengthwise to reveal the fiber layer. This enabled the cross-sections to be conducted along the fibers’ orientation and prevented fiber damage.

## 3. Results

### 3.1. Assessment of Roving Bundle Straightness, Cavities, and Impregnation Condition

**Figure 4:**
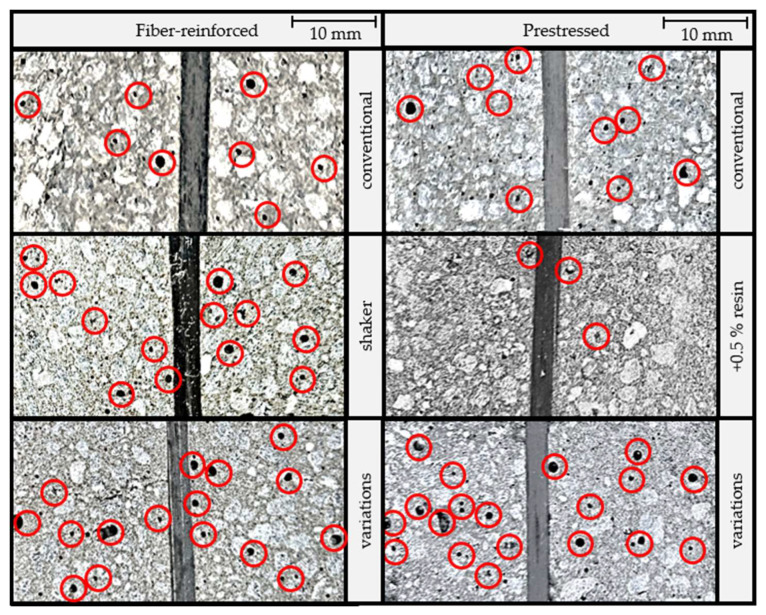
Comparison of the cavities (exemplary marked in red).

**Figure 5:**
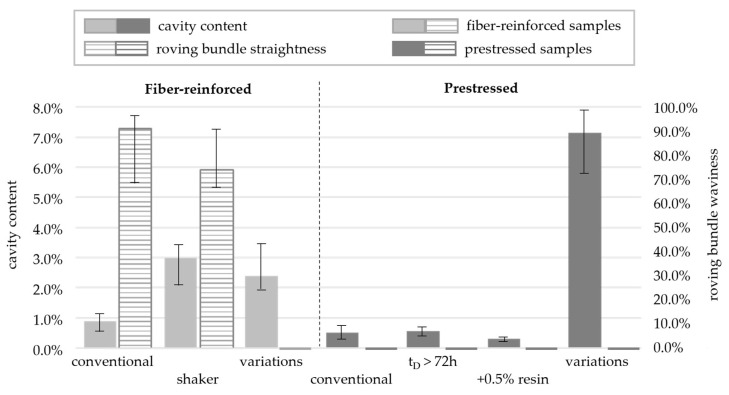
Comparison of cavity content and roving bundle waviness (related to roving bundle width).

**Figure 6:**
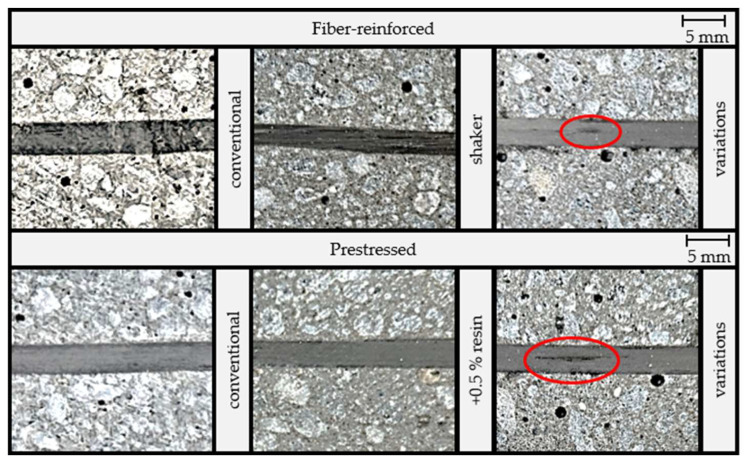
Comparison of the impregnation of the roving bundles (red circles mark dry spots in the rovings).

**Figure 7:**
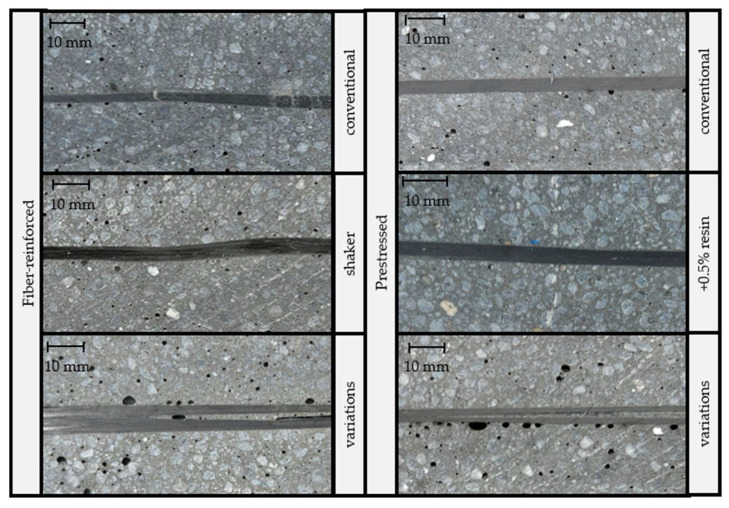
Comparison of the roving bundle alignment and straightness of the samples with the varied process parameters.

The subsequent analysis was focused on the assessment of the samples’ cross-sections, specifically regarding the number of cavities (see Figure 4 and Figure 5), roving bundle matrix saturation (see Figure 6), and roving bundle positioning (see Figure 5 and Figure 7). To streamline the presentation, the samples with extended curing times were omitted as they exhibited similar characteristics to those produced following the procedure outlined in Section 2.1. Moreover, only one sample per variant is shown as a representative example for clarity purposes.

The visual examination revealed a pronounced disparity in the number of cavities between the samples produced with and without the utilization of a shaker. Specifically, the samples subjected to shaking showed a considerably greater number of cavities with a noticeable enlargement of some of these cavities. As initially expected, the use of a higher resin content successfully reduced the formation of cavities. This outcome could be attributed to the lower viscosity observed in the liquid polymer concrete during the manufacturing process. 

The visual results were supported by image evaluation methods. Different percentages of defects were observed depending on the black level of the samples, excluding the roving bundles. The results are shown in Figure 5.

The cavities in the reference samples with fiber reinforcement (no prestressing) amounted to 0.90% of the total area, while the cavities in the samples exposed to the shaker covered 2.97%. The samples with the manufacturing parameters varied according to Table 1 showed a cavity area of 2.40% in the micro-section images. 

Particularly, prestressed samples tended to have fewer cavities, with an area of only 0.50% detected in the reference sample. Furthermore, increasing the resin content reduced the defect percentage to 0.31%. Significant deviations were observed in the prestressed samples with the manufacturing parameters varied according to Table 1. The analysis here revealed that 7.14% of the investigated sample area was covered by cavities.

The comparison of roving bundle impregnations in Figure 6 revealed differences between the two methods employed. Roving bundles impregnated by dipping demonstrate complete resin impregnation. Conversely, in the case of externally applied impregnation, dry spots indicated a localized absence of resin. This discrepancy was particularly evident in the prestressed samples. The results regarding the dry spots in the roving bundle indicated that the dipping method for roving bundle impregnation was advantageous.

The comparison of roving bundle straightness depicted in Figure 7 revealed wavy patterns only in non-prestressed samples, regardless of the shaker application. However, applying a minor prestressing force of 50 N (1.80 MPa) to carbon rovings yielded a straight bundle. This information is further demonstrated in the analysis shown in Figure 5, where the roving bundle waviness *r* was determined through Equation (1) using the width of the roving bundle *w* and the width of the envelope curve around the roving *h*.

(1) $$r=\frac{h-w}{w}$$

However, both samples that underwent variations in the manufacturing process showed a split roving bundle. This was due to the different impregnation methods. The impregnation of the roving bundles with a brush seemed to result in a poorer wetting of the rovings (see Figure 6), which allowed the roving bundle to split.

### 3.2. Influence of Dwell Time in the Mold on Residual Stress Formation

**Figure 8:**
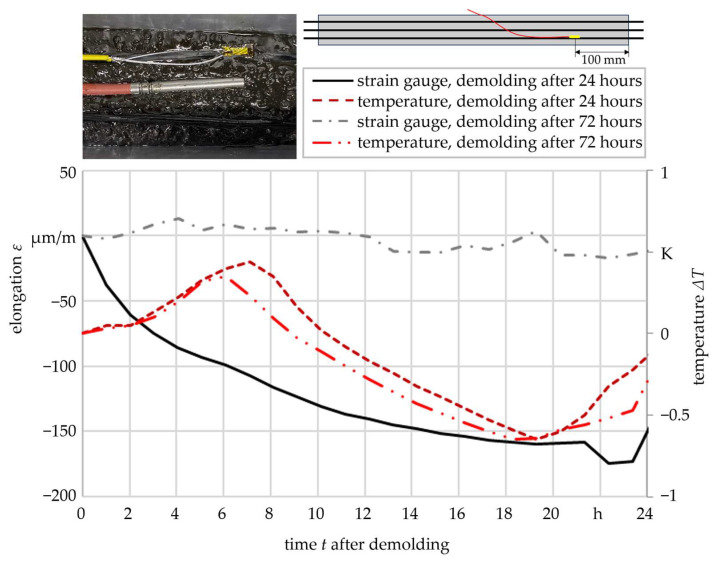
Elongation of integrated strain gauges and temperature in the 24 h after the demolding of the samples cured for 24 h and for 72 h.

To validate the influence of the prestressing force, two test samples with integrated measuring devices (see Figure 1) were manufactured simultaneously in order to be able to neglect the temperature influence during the curing process. One of the samples was demolded after 24 h in accordance with the previous specifications, while the second sample had a dwell time of 72 h in the mold. The prestressing force of the carbon roving bundles was also maintained during the complete dwell time. After the demolding, the strain of the integrated strain gauge and the temperature in the samples were recorded for a further 24 h. Figure 8 shows the results regarding the elongation of the strain gauge and the temperature change during the observation period after demolding for one sample pairing as an example.

The diagram illustrates the significant variation in strain gauge compression during the observation period, depending on the dwell time in the mold. In the case of a demolding after 24 h, the strain gauge showed a substantial compression of more than 170 µm. Conversely, a demolding after 72 h exhibited a minimal change in strain gauge length. Temperature differences could be ruled out as the cause of these disparate strains, as both samples demonstrated a comparable temperature behavior. These results suggested that the previously mentioned material effects made it possible to reduce the tensile stress on the carbon roving bundles, leading to the development of residual compressive stresses within the polymer concrete. Consequently, it was anticipated that samples with an extended dwell time in the mold would yield inferior results in the flexural test.

### 3.3. Assessment of the Bending Properties

In the bending test, all samples were loaded to failure. The strain and the force in the area of elastic deformation were evaluated after the tests. Failure was defined as a reduction in the bending force of 10% or more. The results were used to calculate the stiffness, *S*, on basic of the distance of the point of force application *l*, the force *F*, and the deflection *f* by means of Equation (2).

(2) $$S=\frac{l^{3}}{3}\cdot \frac{F}{f}$$

**Figure 9:**
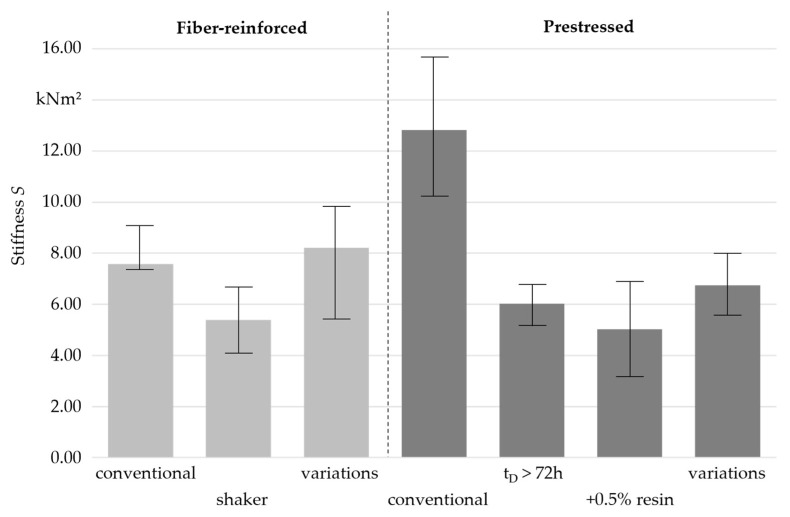
Comparison of the stiffness of the samples with variation in manufacturing parameters (*n* = 3).

The results for the calculated stiffness can be seen in Figure 9. 

Among the fiber-reinforced samples, the samples with varied manufacturing parameters showed the greatest stiffness, likely attributed to the enhanced roving bundle straightness, as described above. However, this also resulted in a smaller range for the stiffness. It was noticed that this non-prestressed sample had a greater stiffness than the prestressed fiber-reinforced samples with the manufacturing parameters varied according to Table 1. This was presumably due to an inadequate impregnation of individual internal roving bundles, resulting from the different impregnation methods (see Figure 6).

As expected, the fiber-reinforced samples exposed to a shaker showed a smaller stiffness compared with the fiber-reinforced samples produced according to the previous specifications (see Section 2.1). This was likely due to an increased number of cavities and the waviness in the fiber reinforcement.

The results suggested a potential for the further development of non-prestressed fiber-reinforced polymer concrete. It remains to be investigated whether a combination of the current manufacturing method and the application of a minor prestressing force can enhance the mechanical properties of fiber-reinforced polymer concrete.

Among the prestressed fiber-reinforced samples, none of them achieved the mechanical properties of the samples produced according to the specifications described in Section 2.1. The results for the samples with an increased resin content were in good agreement with findings in the literature [11]. The reduced stiffness of the samples with varied manufacturing parameters could be attributed to the splitting of the roving bundle, dry spots in the roving bundle, and an increased number of cavities in the polymer concrete.

**Figure 10:**
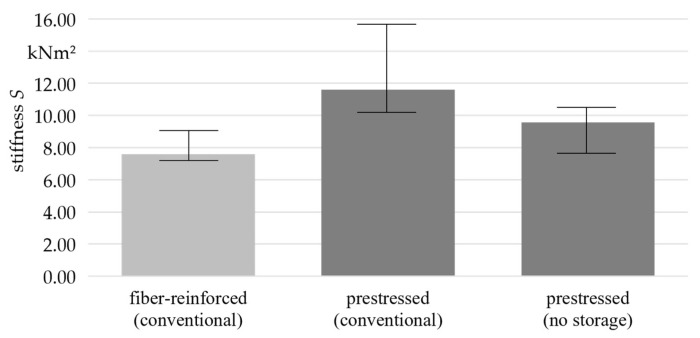
Comparison of the stiffness of prestressed fiber-reinforced polymer concrete manufactured with and without storage time before uniaxial bending test (*n* = 3).

The results for the samples with an increased dwell time in the mold were in good agreement with the assumptions made in Section 2.2, indicating that the formation of residual compressive stresses was hindered by a prolonged dwell time in the mold. To further confirm this assumption, additional prestressed samples were produced according to the specifications in Section 2.1 but subjected to the bending test immediately after demolding. The stiffness results for these samples are presented in Figure 10.

Although the samples tested directly after demolding showed a significantly greater stiffness than the fiber-reinforced non-prestressed samples, they did not achieve the same values like after a storage period. The results confirmed the assumption that the material processes occurring in the first 24 h after demolding are an elementary component in the formation of residual stresses in prestressed fiber-reinforced polymer concrete.

## 4. Discussion

This study was investigating the influences of variations in the manufacturing process of fiber-reinforced polymer concrete and prestressed fiber-reinforced polymer concrete on the large scatter in bending stiffness, determined in previous investigations. Uniaxial bending tests were performed to characterize the samples, and cross-sections of all samples were prepared for analysis.

Regarding pure polymer concrete, it is established in the literature that the mechanical properties can be influenced by the cavity percentage, as described in Section 1. To minimize the number of cavities, fiber-reinforced samples were prepared using shakers to allow outgassing. However, the cross-sections revealed that the use of the shaker resulted in an increased percentage of cavities. Furthermore, the layer of carbon rovings deteriorated. The investigation confirmed the assumption that a higher percentage of cavities in fiber-reinforced polymer concrete results in decreased mechanical properties.

To decrease cavities, the strategy of enhancing polymer concrete viscosity through an increased resin content was employed. However, while this measure can reduce cavity percentages, it also results in a smaller bending stiffness. This effect is similarly apparent in pure polymer concrete [11]. Additionally, this measure did not effectively reduce the large scatter in bending stiffness, suggesting that the proportion of cavities was not the sole contributing factor.

An increase in the dwell time in the mold was the only factor that reduced the scatter of the bending stiffness. The compressive residual stresses arising from the prestressing of the integrated carbon rovings could provide a justification for this. Creep tests and strain monitoring, conducted after demolding, revealed significant material changes within the first 24 h, enabling the formation of residual compressive stresses. It was demonstrated that maintaining the prestressing force beyond the aforementioned 24 h prevented material elongation and, consequently, the formation of residual compressive stresses. These results were validated through the evaluation of mechanical properties in the bending tests.

Furthermore, test samples with variations in more than one manufacturing parameter were analyzed here. These samples revealed that a combination of changes in various manufacturing parameters did not qualify for a reduction in the scatter of bending stiffness either. However, it could be noticed that the application of a minor prestressing force on the reinforcing structure led to an improvement in fiber layer and, thus, also to an increase in bending stiffness. Moreover, the dipping method for roving bundle impregnation was found to be suitable for analyzing the cross-section of these samples. An application of a manual brush transfer resin could, however, result in dry spots within the roving bundles, leading to roving bundle splitting within the sample and a decrease in stiffness.

## 5. Conclusions

Previous studies have shown that the bending stiffness of samples made of prestressed fiber-reinforced polymer concrete, a novel material, exhibits a high degree of scatter when tested in uniaxial bending. It remains uncertain whether the specified manufacturing guidelines are optimized to fully utilize the potential of the material. An analysis of the factors affecting the properties of pure polymer concrete revealed the percentage of cavities and dwell time in the mold to be crucial. This study also demonstrated that these factors significantly affect the production of prestressed fiber-reinforced polymer concrete samples with reproducible flexural properties. Increasing the dwell time in the mold was the only way to improve the reproducibility of the bending properties. However, as this factor led to a decrease of 53% in bending stiffness, a variation of this factor is strongly discouraged. The analysis of the cross-sections revealed important findings regarding the manufacturing process. Specifically, the use of a shaker during the production of the polymer concrete EPUMENT 130/3 is discouraged, as it led to an increase of 230% in the number and size of cavities. Furthermore, the cross-section analysis revealed that it is imperative to prestress the roving bundle reinforcement to create a uniform fiber layer. A minor prestressing force of 50 N is sufficient to achieve this objective.

In summary, deviations from the established production method were found to be disadvantageous, particularly for prestressed fiber-reinforced polymer concrete. For fiber-reinforced polymer concrete, however, the application of a minor prestressing force during the manufacturing process showed a potential for further development.

## Figures and Tables

**Table 1:** Test plan for analyzing the influence of different manufacturing parameters on cavities, roving bundle straightness, roving bundle impregnation, and stiffness in fiber-reinforced and prestressed fiber-reinforced polymer concrete.

		Prestressing force (N)	Shaker usage	Resin content (wt%)	Dwell time in the mold (h)	Impregnation method for the roving
**Fiber-reinforced samples**	According to Figure 1 (“conventional”)	0	No	10.8	24	Dipping
	With shaker (“shaker”)	0	Yes	10.8	24	Dipping
	Defined variations (“variations”)	50	Yes	10.8	24	Manual (brush)
**Prestressed samples**	According to Figure 1 (“conventional”)	3000	No	10.8	24	Dipping
	Higher resin percentage(“+0.5% resin”)	3000	No	11.3	24	Dipping
	Dwell time in the mold > 72 h (“t_D_ > 72 h”)	3000	No	10.8	72	Dipping
	Defined variations (“variations”)	2000	Yes	10.8	24	Manual (brush)

## Data Availability

Data are contained within the article.
